# Development and psychometric validation of the insect fear questionnaire for school-aged children in Iran

**DOI:** 10.1371/journal.pone.0344126

**Published:** 2026-03-06

**Authors:** Arman Latifi, Mostafa Farahbakhsh, Saideh Yousefi, Somayeh Azimi, Mahasti Alizadeh, Tohid Jafari-Koshki, Aboozar Soltani, Sara Rahimi, Madineh Abbasi

**Affiliations:** 1 Research Center for Evidence-Based Health Management, Maragheh University of Medical Sciences, Maragheh, Iran; 2 Research Center of Psychiatry and Behavioral Sciences, Tabriz University of Medical Sciences, Tabriz, Iran; 3 Department of Public Health, Sirjan School of Medical Sciences, Sirjan, Iran; 4 Social Determinants of Health Research Center, Yasuj University of Medical Sciences, Yasuj, Iran; 5 Medical Education Research Center, Health Management and Safety Promotion Research Institute, Tabriz University of Medical Sciences, Tabriz, Iran; 6 Department of Community and Family Medicine, Faculty of Medicine, Tabriz University of Medical Sciences, Tabriz, Iran,; 7 Molecular Medicine Research Center, Tabriz University of Medical Sciences, Tabriz, Iran; 8 Department of Statistics and Epidemiology, Faculty of Health, Tabriz University of Medical Sciences, Tabriz, Iran; 9 Research Center for Health Sciences, Institute of Health, Department of Medical Entomology and Vector Control, School of Health, Shiraz University of Medical Sciences, Shiraz, Iran; 10 Medicinal Plants Research Center, Maragheh University of Medical Sciences, Maragheh, Iran; 11 Infectious and Tropical Diseases Research Center, Tabriz University of Medical Sciences, Tabriz, Iran; Khoy University of Medical Sciences, IRAN, ISLAMIC REPUBLIC OF

## Abstract

**Background:**

Insect-related fear (Entomophobia) is prevalent in children and affects their behavior and well-being, avoidance behaviors, and potentially hazardous practices such as inappropriate pesticide use. While many tools exist for measuring fear in adults, there is a lack of validated instruments for school-aged children. This study aimed to develop and validate a questionnaire assessing knowledge, entomophobia, behavior, and anxiety about insects among school-aged children in East Azerbaijan, Iran.

**Methods:**

This psychometric study involved a multi-stage stratified random sampling design in East Azerbaijan Province, Iran. A researcher-made questionnaire, developed through expert consultation and literature review, was used. A total of 1,370 children participated. Content validity was quantified via CVR and CVI. Construct validity was assessed with exploratory factor analysis (EFA) and confirmatory factor analysis (CFA); EFA and reliability analyses (Cronbach’s alpha, item-total statistics) were performed in IBM SPSS Statistics 25, and CFA was conducted in AMOS 22. Temporal stability was evaluated by test–retest Intraclass Correlation Coefficients (ICC) over a two-week interval (n = 103).

**Result:**

The finalized instrument comprises 32 items loading on four factors: knowledge, entomophobia, behavior, and personal fear/anxiety. EFA and CFA supported the four-factor model with excellent fit (CFI = 0.96, TLI = 0.95, RMSEA = 0.047, SRMR = 0.041). Internal consistency was acceptable (Cronbach’s α = 0.82), and test–retest reliability indicated good temporal stability (ICC = 0.81; 95% CI: 0.76–0.86). No meaningful floor or ceiling effects were observed.

**Conclusion:**

The questionnaire demonstrates sound psychometric properties and is suitable for use in school settings to identify children with elevated insect-related fear or unsafe pesticide practices. Future work should examine criterion validity, measurement invariance across subgroups, and applicability in other cultural contexts.

## Introduction

Specific phobias are a subtype of anxiety disorders characterized by intense and disproportionate fear toward specific objects or situations, often emerging during childhood and capable of influencing emotional, cognitive, and behavioral development if left untreated [1,2]. Among these, insect-related fears, including entomophobia and arachnophobia, are particularly common in school-aged children and arise from complex interactions among biological predispositions, cognitive processes, learning experiences, and sociocultural influences [3–5].

Empirical data indicate that animal-related phobic reactions typically emerge between ages 10 and 13, a period marked by the ongoing development of emotional regulation and neural circuits involved in fear processing [6]. Although these fears may seem insignificant to adults, they can cause considerable distress for children. Since insects and spiders are pervasive in both urban and natural environments, repeated encounters can intensify children’s phobic responses [7–10]. Crucially, untreated specific phobias often manifest as avoidance and social withdrawal, which can significantly diminish children’s engagement in outdoor education, scientific exploration, and leisure activities [11].

From an epidemiological perspective, roughly 6.2% of the population experiences a range of phobic symptoms, with approximately 1% enduring severe phobias that can seriously disrupt daily life [10,12]. Research regarding arachnophobia specifically estimates that it affects between 2.7% and 6.1% of the general population [3,8,13,14].

A cross-sectional study in Iran involving 531 school-aged children found that 35.8% reported a fear of insects or arachnids, while 52.5% indicated disgust at their sight [10]. Moreover, 33.3% exhibited moderate phobic symptoms, and 4.5% displayed signs of severe phobia. These findings highlight the public health significance of insect and spider-related phobias during childhood and adolescence [10,15]. Consistent with global data, research demonstrates significant gender differences in prevalence, with higher rates consistently observed among girls [16–20]. For instance, Iranian research has reported phobia rates up to six times greater in girls [10], while a Czech population-based survey found a female to male ratio of 4:1 for spider phobia [9]. Explanatory frameworks for these differences draw on higher disgust sensitivity, gendered socialization patterns, evolutionary biology, and developmental psychology [16–20]. Furthermore, research on the evolutionary origins of insect-related fears, alongside studies of genetic mechanisms, underscores how parental conduct most notably maternal behavior can foster fearfulness in children [19,21].

The assessment of these fears in children requires developmentally appropriate and psychometrically sound instruments. Previous research has established the distinct structure of specific phobia symptoms in children, confirming that subtypes such as animal phobias are measurable constructs [22]. Several general instruments have been developed to assess these fears. For example, the Revised Fear Survey Schedule for Children (FSSC-R) has demonstrated utility in discriminating among different types of phobias, including those related to small animals [23]. Similarly, the Koala Fear Questionnaire (KFQ) offers a standardized self-report scale using visual cues suitable for pre-school and primary school children [24]. While these instruments provide a valuable broad overview of childhood fears, they are designed as general screening tools and often lack the granularity required to assess the specific cognitive and behavioral dimensions of insect-related fear. Conversely, more specific tools such as the Spider Phobia Questionnaire for Children (SPQ-C) have been validated to assess spider fear specifically [25]. However, the SPQ-C focuses exclusively on arachnids and does not encompass the broader category of insects, nor does it capture context-specific behavioral responses, such as the use of pesticides, which are relevant in many cultural settings.

Currently, robust psychometric instruments specifically designed to measure the full spectrum of insect-related fears in school-age populations remain limited. While existing measures such as the Spider Phobia Questionnaire (SPQ), the Spider Beliefs Questionnaire (SBQ), and the Original Adults Entomophobia and Arachnophobia Scale (OAEAS) have demonstrated high reliability and validity [9,10,15], they are primarily validated for adult populations. These adult-oriented instruments are not developmentally suitable for children due to distinct differences in emotional processing, cognitive maturity, and language comprehension. The lack of a validated, child-specific tool that address insect-related fears limits diagnostic accuracy, hinders cross-study comparisons, and impedes the development of early intervention programs. Furthermore, because insect-related fears may be expressed differently across cultural and ecological environments, there is a critical need for tools adapted to specific populations.

To accommodate the multifaceted nature of children’s responses to insects, the present study adopted a multidimensional conceptual framework. Drawing on theoretical perspectives from environmental psychology, cognitive–behavioral models of fear and phobia [26], and literature on children’s eco-behavioral tendencies, the constructs of knowledge, general fear, insect-related fears, pesticide-related behavior, and situational anxiety were conceptualized as distinct but interrelated domains. This framework guided the definition of the questionnaire’s subscales and ensured that item development addressed both general and specific dimensions of insect-related fear. Therefore, the present study aimed to develop and validate a comprehensive questionnaire to measure knowledge, insect-related fear, anxiety, and behavioral practices concerning insects and spiders among students in Iran.

## Materials and methods

### Study design and participants

This was methodological research of cross-sectional design, and its purpose was to create and psychometrically test a questionnaire to measure school children’s insect-related fear. A multi-stage stratified random sampling method was used to ensure representation from different educational levels and geographic regions of East Azerbaijan Province. First, all counties in the province (23 counties) were identified. In the first stage, schools were randomly selected from each county proportionate to the children population size (PPS sampling). In the second stage, within each selected school, classes were chosen through simple random sampling. In the final stage, students within each class were randomly selected using a random number table. This procedure ensured unbiased and proportional allocation of participants across counties and educational levels (primary, secondary, and post-secondary). Preliminary sample size was computed as 1067 to achieve margin error of 0.03 for estimating response proportion of 0.5 with 95% level of confidence. By taking the design effect of 1.2 for clustered sampling plan and attrition rate of 10% into account, the final sample size was calculated as 1408. Of these, 1,370 children completed the questionnaires in full, resulting in a participation rate of 97.3%. Inclusion criteria were currently enrollment in day schools in the province and supplying informed consent (and parental consent in minors) to participate. Children were excluded if they had a diagnosed psychological or developmental disorder, provided incomplete responses, or withdrew consent. Questionnaires with patterned or invalid responses were also excluded from analysis.

### Data collection procedure

Data were collected in classrooms during regular school hours to ensure a standardized environment. The questionnaires were administered in paper form by trained research assistants who were not part of the teaching staff and had no supervisory relationship with the children. This approach minimized potential bias and reduced pressure on children to participate. Teachers were present only to maintain classroom order and did not influence the data collection process. Children completed the questionnaires individually and anonymously, without discussing their answers with peers. For the test–retest reliability subsample, the same procedure was repeated two weeks later following identical instructions. Completed questionnaires were sealed immediately and transferred securely to the research team for data entry and analysis. The data were collected between October 2024 and May 2025 in the form of a structured questionnaire.

### Ethical considerations

This study was approved by the Ethics Committee of Tabriz University of Medical Sciences (Approval Code: IR.TBZMED.REC.1402.536). Before data collection, written informed consent was obtained from parents or legal guardians, and verbal assent was obtained from all participating children. Information about the study including, its purpose, voluntary nature, confidentiality of responses, and the right to withdraw at any stage, was communicated to parents and children through official letters distributed by the schools. Teachers explained the study information verbally to children in age-appropriate language to ensure understanding. children were given time to ask questions, and only those who clearly expressed a willingness to participate were included.

### Instrument development

The development of the questionnaire followed a theory-driven, multidimensional approach based on established guidelines for scale construction [27]. Before item generation, four conceptual domains were defined: [1] knowledge about insects, [2] insect-related fear, [3] pesticide-related behavior, and [4] personal fear and anxiety. These domains were derived from cognitive–behavioral theories of insect-related fear, environmental health models, and prior instruments assessing insect-related attitudes. Items for each domain were generated separately to avoid conceptual overlap. General fear/anxiety items assessed emotional responses in everyday contexts, whereas phobia-specific items focused on intense reactions and avoidance tendencies characteristic of insect-related fear. Behavioral items addressed pesticide use, while knowledge items reflected factual understanding. This domain-based structure ensured that questions measuring general anxiety did not confound those targeting specific phobic reactions.

The questionnaire consisted of four constructs: knowledge (4 items, e.g., I can easily identify insects and spiders), entomophobia (19 items, e.g., I get scared if I see an insect or a spider), pesticide use behavior (4 items, e.g., I use insect repellent spray or gel on my skin), and personal fear and anxiety (5 items, e.g., I am a worrier and feel anxious about everything). Items were developed through a two-stage process. First, an initial pool was created using a comprehensive literature review and adaptation of items from validated instruments [10,28,29], supplemented with newly developed items by the research team. Second, a panel of 15 experts evaluated the items for relevance, clarity, and cultural suitability. Items not meeting predefined criteria (adequate relevance, clarity, non-redundancy, and age-appropriateness) or with low CVR/CVI scores were revised or removed. This iterative process resulted in the final set of 32 items used in the study. Items were framed in five-point Likert format: knowledge and entomophobia: 1 = Strongly disagree to 5 = Strongly agree. Pesticide use and personal anxiety: 1 = Always to 5 = Never. For negatively worded items, reverse scoring was applied to ensure that higher total scores consistently reflected higher levels of the respective construct.

### Face validity

Qualitative face validity was established via a 15-member expert panel of subject-matter specialists in medical entomology, health education, methodology, and psychology. Experts reviewed items one-on-one for ease of wording, grammatical adequacy, and sensitivity to cultural awareness. Quantitative face validity was established based on item impact scores, and an impact score of ≥ 1.5 was accepted.

### Content validity

Content validity was evaluated using both the Content Validity Ratio (CVR) and the Content Validity Index (CVI). CVR was computed based on Lawshe’s method, with a minimum acceptable value of 0.49 for 15 experts [30]. Items with CVI values ≥ 0.78 were retained. The S-CVI/Average of ≥ 0.90 were considered acceptable.

### Pilot testing

A pilot study was conducted with 30 children selected through the same multi-stage stratified sampling strategy used in the main study, ensuring representativeness in grade level and school context. children from one urban and one rural school participated voluntarily with parental consent and children assent. The pilot data were used exclusively to refine item wording and assess comprehension. There were no significant adjustments needed after the pilot.

### Construct validity

#### Exploratory factor analysis (EFA).

EFA of the main study sample (n = 1,370) responses employed Principal Axis Factoring to investigate underlying factor structure. The Kaiser–Meyer–Olkin (KMO) statistic determined sampling adequacy, and values ≥0.60 were accepted as acceptable. Bartlett’s Test of Sphericity validated factorability (p < 0.05). Extraction of factors employed the eigenvalue >1 criterion and visual examination of the scree plot. The items underwent varimax rotation to facilitate interpretability. Items that had loading ≥0.40 on their respective factors and lacked high cross-loadings were retained.

### Confirmatory factor analysis (CFA)

CFA was performed using AMOS version 22 with the maximum likelihood estimation method to verify the factor structure identified in the EFA. Model fit was evaluated using the chi-square to degrees of freedom ratio (χ²/df), Comparative Fit Index (CFI), Tucker–Lewis Index (TLI), Root Mean Square Error of Approximation (RMSEA) with 90% confidence intervals, and the Standardized Root Mean Square Residual (SRMR). Acceptable model fit was defined as χ²/df ≤ 5, CFI ≥ 0.90, TLI ≥ 0.90, RMSEA ≤0.08, and SRMR ≤0.08. Standardized factor loadings were examined, and items with loadings ≥ 0.40 were retained. Modification indices were reviewed, and correlated error terms were added only when theoretically justified

### Reliability

Internal consistency reliability was examined through Cronbach’s alpha, and levels ≥0.70 were accepted as suitable. The test–retest reliability of a random selection of 103 children was determined by the Intraclass Correlation Coefficient (ICC, two-way mixed-effects model, absolute agreement). ICC levels ≥0.75 were found to denote good reliability.

### Missing data management

Before they were analyzed, the dataset had been checked for missing values. The rate of missing responses for each item was lower than 5%, which is typically regarded as negligible in psychometric research. Due to the low incidence and sporadic missingness pattern, missing responses were treated utilizing the Expectation–Maximization (EM) algorithm to best use available information without bias. All subsequent analyses were conducted on the imputed dataset

### Statistical analysis

Descriptive statistics (mean, standard deviation, skewness, and kurtosis) were computed for every item. Pearson correlation coefficients were calculated to examine the association between age and the scores of the four constructs (knowledge, insectophobia, pesticide-related behavior, and personal fear and anxiety). Independent-samples t-tests were used to compare construct scores between male and female children. Ceiling and floor effects were examined, and >15% of the responses at the minimum or maximum score were judged to be indicative of effects of this type. All the analyses were conducted on IBM SPSS Statistics version 25 for descriptive statistics, EFA, and reliability, and on AMOS version 22 for CFA.

## Results

### Participants

A total of 1370 children participated in the study, with a mean age of 13.85 (ranged from 6 to 18) years (SD = 3.33). Table 1 presents the demographic characteristics of the participants.

**Table 1:** Demographic characteristics of participants.

Variable	Category	n	%
**Gender**	Male	439	32
	Female	931	68
**Location**	Urban	1265	92.3
	Rural	105	7.7
**Grade level**	Primary	464	33.9
	Secondary	348	25.4
	Post-secondary	558	40.7
**Type of school**	Public	924	69
	Private	425	31

### Face and content validity

All items had an impact score ≥ 1.5, which validated acceptable face validity. The CVR in content validity analysis varied between 0.6 and 1, all of which were higher than the minimum standard of 0.49 for 15 experts. The I-CVI varied between 0.8 and 1, which met the minimum acceptable criterion of ≥ 0.78. The S-CVI/Average was 0.96, which shows high content validity. None of the items were removed according to these findings (Table 2).

**Table 2:** Content validity results for each item (n = 15 experts).

Item no.	CVR	I-CVI	Decision
**Q1**	1	1	Retained
**Q2**	1	0.93	Retained
**Q3**	1	1	Retained
**Q4**	1	1	Retained
**Q5**	1	1	Retained
**Q6**	0.86	0.93	Retained
**Q7**	1	1	Retained
**Q8**	0.73	0.93	Retained
**Q9**	1	1	Retained
**Q10**	1	0.93	Retained
**Q11**	1	1	Retained
**Q12**	1	0.93	Retained
**Q13**	1	1	Retained
**Q14**	1	1	Retained
**Q15**	1	1	Retained
**Q16**	1	1	Retained
**Q17**	1	1	Retained
**Q18**	1	0.93	Retained
**Q19**	1	0.93	Retained
**Q20**	1	1	Retained
**Q21**	1	1	Retained
**Q22**	1	1	Retained
**Q23**	0.60	0.80	Retained
**Q24**	1	1	Retained
**Q25**	1	1	Retained
**Q26**	0.86	0.86	Retained
**Q27**	1	0.93	Retained
**Q28**	1	0.93	Retained
**Q29**	1	1	Retained
**Q30**	1	0.93	Retained
**Q31**	0.86	0.93	Retained
**Q32**	1	0.93	Retained

Note: CVR ≥ 0.49 and I-CVI ≥ 0.78 considered acceptable; S-CVI/Ave ≥ 0.90 indicates excellent content validity.

### Pilot testing

Pilot study (n = 30) found the mean completion time to be five minutes. There were no significant comprehension problems, and suitable response distributions were found for all items.

### Construct validity

#### Exploratory factor analysis.

KMO statistic was 0.95, which reflected sampling adequacy, and Bartlett’s Test of Sphericity was significant (χ² = 24002.23, df = 496, p < 0.000), which validated the data for factor analysis. EFA identified 4 factors, which explained 60.9% of total variance. Factor loading of retained items varied between 0.19 and 0.98 (Table 3). There were no significant cross-loadings, and all retained items loaded high on their respective factors.

**Table 3:** Rotated factor loading matrix for the questionnaire (EFA).

Item no.	Factor 1 (Knowledge)	Factor 2 (Entomophobia)	Factor 3 (Behavior)	Factor 4 (Personal Fear and Anxiety)
**Q1**	**0.75**			
**Q2**	**0.70**			
**Q3**	**0.81**			
**Q4**	**0.69**			
**Q5**		**0.72**		
**Q6**		**0.75**		
**Q7**		**0.79**		
**Q8**		**0.68**		
**Q9**		**0.56**		
**Q10**		**0.64**		
**Q11**		**0.61**		
**Q12**		**0.78**		
**Q13**		**0.79**		
**Q14**		**0.65**		
**Q15**		**0.81**		
**Q16**		**0.77**		
**Q17**		**0.74**		
**Q18**		**0.66**		
**Q19**		**0.63**		
**Q20**		**0.77**		
**Q21**		**0.78**		
**Q22**		**0.69**		
**Q23**		**0.57**		
**Q24**			**0.82**	
**Q25**			**0.74**	
**Q26**			**0.69**	
**Q27**			**0.79**	
**Q28**				**0.76**
**Q29**				**0.80**
**Q30**				**0.71**
**Q31**				**0.69**
**Q32**				**0.70**

**Note:** Extraction method: Principal Axis Factoring; Rotation: Varimax; Loadings ≥ 0.40 retained.

**Fig 1:**
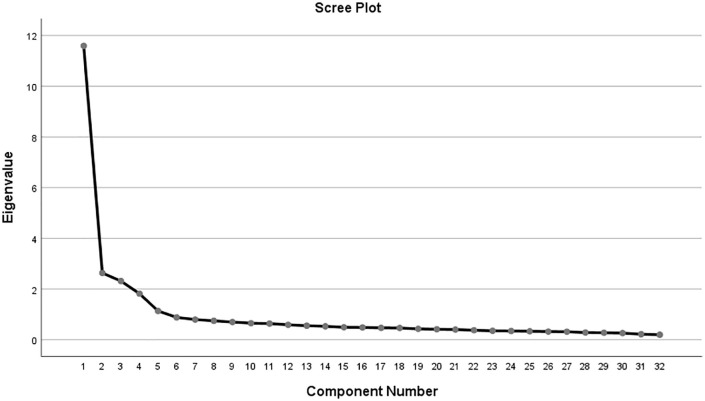
Scree plot. Created originally by the authors using IBM SPSS AMOS 22.

A scree plot (Fig 1) was studied in order to ascertain the best number of factors to retain. In the current research, the scree plot favored retention of four factors, similar to the result obtained based on the eigenvalue >1 criterion and theoretical arguments. The four-factor solution corresponds well to the postulated structure of the questionnaire, which had been developed to measure: Knowledge (4 items), Entomophobia (19 items), Pesticide Use (4 items), and, Personal Fear and Anxiety (5 items). The matching of empirical criteria of factor retention and theoretical construct strengthens the construct validity of the tool and provides justification for its use in measuring specific but interrelated aspects of insect-associated fear in children.

### Confirmatory factor analysis

The CFA confirmed the factor structure determined in the EFA. The model had acceptable fit: χ²/df 2.99 (p < 0.001), CFI = 0.96, GFI = 0.93, TLI = 0.95, RMSEA = 0.047, and SRMR = 0.041, and exceeded recommended cut-offs (Table 4). All of the standardized factor loadings were ≥ 0.40, and ranged from 0.46 to 0.85, and were significant statistically (p < 0.05) (Table 5).

**Table 4:** Fit indices of the confirmatory factor analysis model.

Indices	Acceptable values	Fit index in the CFA model	Interpretation
**χ2, df, p-value**	p > 0.05	χ2 = 1372.80, df = 458, p = 0.000	–
**χ2/df**	≤ 5	2.99	Good fit
**CFI**	≥ 0.90	0.96	Excellent fit
**GFI**	>0.90	0.93	Good fit
**TLI**	≥ 0.90	0.95	Excellent fit
**RMSEA**	≤ 0.08 (≤ 0.05 ideal)	0.047	Close fit
**SRMR**	≤ 0.08	0.041	Good fit

**Note:** χ2(df): chi-square statistics (degree of freedom), TLI: Tucker–Lewis index, CFI: comparative fit index, GFI: goodness of fit index, SRMR: standardized root mean square residual, RMSEA: root mean square error of approximation.

**Table 5:** Standardized factor loadings from CFA.

Item no.	Factor	Standardized Loading	p-value
Q**1**	**Knowledge**	**0.81**	**<0.001**
**Q2**	**Knowledge**	**0.62**	**<0.001**
**Q3**	**Knowledge**	**0.47**	**<0.001**
**Q4**	**Knowledge**	**0.51**	**<0.001**
**Q5**	**Entomophobia**	**0.75**	**<0.001**
**Q6**	**Entomophobia**	**0.69**	**<0.001**
**Q7**	**Entomophobia**	**0.62**	**<0.001**
**Q8**	**Entomophobia**	**0.64**	**<0.001**
**Q9**	**Entomophobia**	**0.85**	**<0.001**
**Q10**	**Entomophobia**	**0.70**	**<0.001**
**Q11**	**Entomophobia**	**0.75**	**<0.001**
**Q12**	**Entomophobia**	**0.72**	**<0.001**
**Q13**	**Entomophobia**	**0.82**	**<0.001**
**Q14**	**Entomophobia**	**0.63**	**<0.001**
**Q15**	**Entomophobia**	**0.78**	**<0.001**
**Q16**	**Entomophobia**	**0.68**	**<0.001**
**Q17**	**Entomophobia**	**0.84**	**<0.001**
**Q18**	**Entomophobia**	**0.66**	**<0.001**
**Q19**	**Entomophobia**	**0.69**	**<0.001**
**Q20**	**Entomophobia**	**0.80**	**<0.001**
**Q21**	**Entomophobia**	**0.76**	**<0.001**
**Q22**	**Entomophobia**	**0.81**	**<0.001**
**Q23**	**Entomophobia**	**0.46**	**<0.001**
**Q24**	**Behavior**	**0.57**	**<0.001**
**Q25**	**Behavior**	**0.53**	**<0.001**
**Q26**	**Behavior**	**0.63**	**<0.001**
**Q27**	**Behavior**	**0.58**	**<0.001**
**Q28**	**Personal fear and Anxiety**	**0.77**	**<0.001**
**Q29**	**Personal fear and Anxiety**	**0.78**	**<0.001**
**Q30**	**Personal fear and Anxiety**	**0.62**	**<0.001**
**Q31**	**Personal fear and Anxiety**	**0.71**	**<0.001**
**Q32**	**Personal fear and Anxiety**	**0.74**	**<0.001**

**Note:** Loadings ≥ 0.40 retained; all p-values < 0.05 indicate statistical significance.

**Fig 2:**
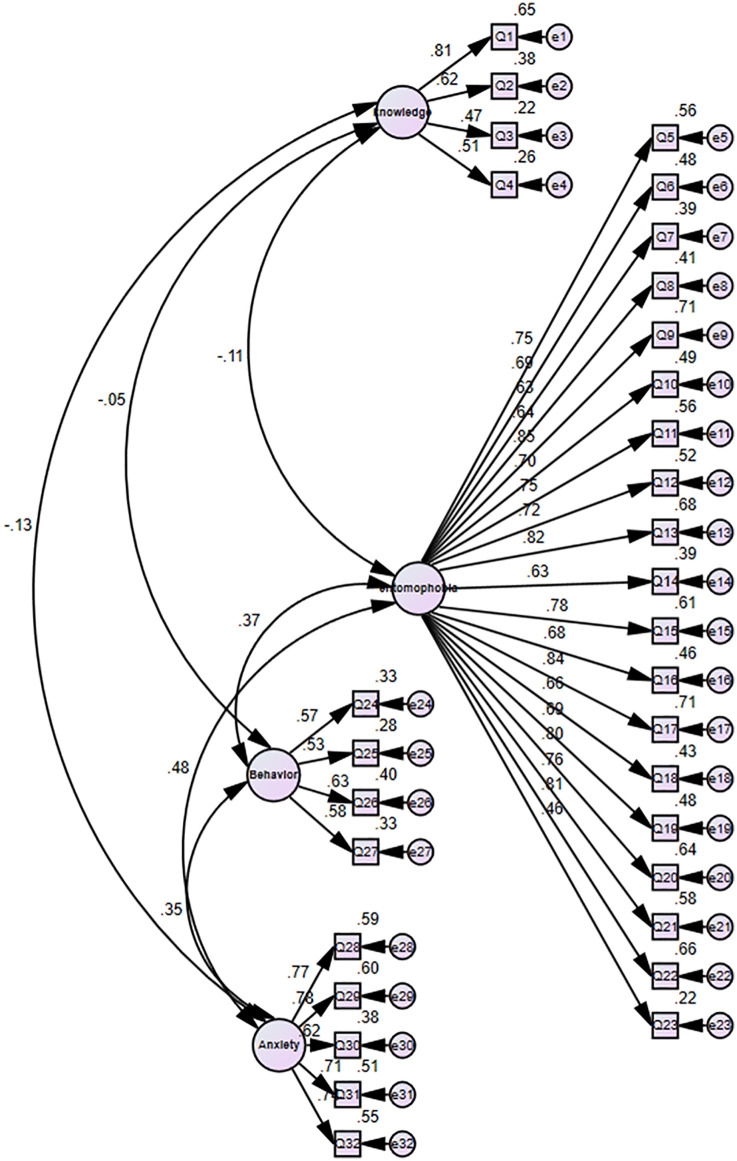
Path diagram of the confirmatory factor analysis model. Created originally by the authors using IBM SPSS AMOS 22.

The path diagram of CFA (Fig 2) shows the associations of the four latent variables and their corresponding observed indicators. All of the standardized factor loadings were statistically significant (p < 0.05) and were higher than 0.40, and they ranged from 0.46 to 0.85.

### Internal consistency reliability

The Cronbach’s alpha coefficients indicated good to excellent internal consistency for all subscales, which ranged from 0.73 to 0.91. The overall scale had a Cronbach’s alpha of 0.82, which represents high reliability. The results of internal consistency are presented in Table 6.

**Table 6:** Internal consistency reliability (Cronbach’s alpha) & Test–retest reliability (Intraclass Correlation Coefficient).

Subscale	No. of item	α	ICC (95% CI)
**Knowledge**	4	0.86	0.85 (0.79-0.90)
**Entomophobia**	19	0.91	0.90 (0.87-0.93)
**Behavior**	4	0.73	0.72 (0.63-0.80)
**Personal fear and Anxiety**	5	0.78	0.77 (0.69-0.84)
**Total**	32	0.82	0.81 (0.76-0.86)

### Test–retest reliability

Reliability analysis of test–retest in the subsample (n = 103) showed fair stability during the two-week period, and ICC ranged from 0.72 to 0.90. The overall scale ICC was 0.81 (95% CI:0.76–0.86). (Table 6).

### Descriptive statistics and ceiling/floor effects

Range of items ranged from 1.18 to 4.06, and standard deviations of items ranged from 0.62 to 1.47. The skewness and kurtosis were in the acceptable range (± 2), revealing rough normality of distribution. The proportion of responses at extreme (either minimum or maximum) value was below 15 in all items, which means there were no severe ceiling and floor effects (Table 7).

**Table 7:** Descriptive statistics, skewness, kurtosis, and ceiling/floor effects for each item (n = 1,370).

Item no.	Possible range	Means (SD)	Skewness	Kurtosis	Ceiling/Floor Effect
Q1. I can easily identify insects and spiders.	1-5	3.97 (1.12)	−1.02	0.34	No
Q2. I am familiar with how insects and spiders live.	1-5	3.32 (1.10)	−0.30	−0.48	No
Q3. Insects and spiders are useful creatures in nature.	1-5	3.46 (1.23)	−0.51	−0.67	No
Q4. Insects and spiders cause allergies in some people.	1-5	4.06 (1.04)	−1.27	1.29	No
Q5. I get scared if I see an insect or a spider.	1-5	3.54 (1.44)	−0.58	−1.08	No
Q6. If I see an insect or a spider, I think it will jump on me.	1-5	3.62 (1.30)	−0.62	−0.80	No
Q7. I am afraid of insect and spider bites.	1-5	3.93 (1.21)	−1.11	0.25	No
Q8. I hate insects and spiders.	1-5	3.67 (1.27)	−0.60	−0.75	No
Q9. Insects and spiders are one of my worst fears.	1-5	3.23 (1.36)	−0.19	−1.25	No
Q10. I feel uncomfortable if I see a picture of an insect or a spider.	1-5	2.91 (1.40)	0.08	−1.30	No
Q11. If I see an insect or a spider, I can hardly stay calm.	1-5	3.09 (1.44)	−0.07	−1.39	No
Q12. I’m worried that there are a lot of insect and spider nests around me.	1-5	3.27 (1.40)	−0.28	−1.22	No
Q13. I get worried if someone says there are insects or spiders around me.	1-5	3.49 (1.34)	−0.52	−0.99	No
Q14. I am very worried that I may be allergic to insect and spider bites or stings.	1-5	3.44 (1.30)	−0.46	−0.91	No
Q15. If I hear the sound of an insect, I become extremely anxious.	1-5	3.25 (1.33)	−0.22	−1.17	No
Q16. If I see an insect or a spider, I sweat with fear.	1-5	2.68 (1.37)	0.33	−1.13	No
Q17. If I see an insect or a spider, I run away.	1-5	3.35 (1.44)	−0.33	−1.28	No
Q18. Before I enter a room, I check to make sure there are no insects or spiders there.	1-5	2.83 (1.44)	0.15	−1.34	No
Q19. If I see an insect or a spider, I can’t get it out of my mind for a long time.	1-5	2.89 (1.47)	0.10	−1.40	No
Q20. If I have seen insects or spiders in a room before, I am worried about entering that room.	1-5	3.24 (1.45)	−0.29	−1.33	No
Q21. If I see an insect or a spider, I ask someone to kill it.	1-5	3.53 (1.45)	−0.60	−1.06	No
Q22. If I see an insect or spider in a room, I quickly leave the room.	1-5	3.47 (1.41)	−0.47	−1.14	No
Q23. Even if there are insects and spiders in the neighbor’s house, I will do my best to get rid of them.	1-5	2.60 (1.34)	0.38	−1.04	No
Q24. I use insect repellent spray or gel on my skin.	1-5	1.91 (1.18)	1.09	0.17	No
Q25. I use insecticides at home.	1-5	2.96 (1.25)	0.04	−0.90	No
Q26. I use insecticides at school.	1-5	1.37 (0.81)	2.00	1.97	No
Q27. I have insecticide with me.	1-5	1.18 (0.62)	1.96	1.90	No
Q28. I am a worrier and feel anxious about everything.	1-5	2.86 (1.31)	0.12	−1.04	No
Q29. When I have a problem, I get a strange feeling in my heart.	1-5	3.39 (1.27)	−0.33	−0.90	No
Q30. I am a fearful person.	1-5	2.38 (1.23)	0.48	−0.74	No
Q31. Whenever I am worried or stressed, my heart beats fast.	1-5	3.53 (1.27)	−0.43	−0.88	No
Q32. I’m worried that something bad will happen to me.	1-5	2.71 (1.36)	0.29	−1.08	No

### Age- and gender-related analyses

To explore the potential influence of demographic variables on the construct scores, supplementary analyses were conducted. Pearson correlation analyses indicated that age demonstrated a very small positive correlation with knowledge (r = 0.10, p < 0.001) and anxiety (r = 0.077, p = 0.004), while showing no significant associations with insectophobia (r = 0.035, p = 0.199) or pesticide-related behavior (r = 0.009, p = 0.752). Although two associations reached statistical significance, all effect sizes were negligible, indicating no meaningful developmental influence on the constructs. Independent-samples t-tests were performed to compare construct scores between male and female participants. No statistically significant gender differences were observed in knowledge, behavior, or anxiety (all p > .05). A small difference was detected in insectophobia scores, with females scoring slightly higher; however, the effect size was minimal (Cohen’s d < 0.20), suggesting that this difference was not substantial enough to bias the psychometric results. Overall, these findings indicate that neither age nor gender meaningfully affected the construct scores or the validity of the instrument.

## Discussion

This work intended to design and validate a general questionnaire for assessing knowledge, entomophobia, pesticide behavior, and personal anxiety for school-aged children from East Azerbaijan Province. The findings indicated that the measure possesses strong psychometric properties on numerous fronts, including content validity, construct validity, internal consistency, and temporal stability, and sensitivity without floor and ceiling effects, which are consistent with internationally accepted best practices for designing and validating instruments [31,32].

The scale content validity was also substantiated with the rating of 15 subject matter experts. Both the content validity ratio (CVR) and content validity index (CVI) exceeded Lawshe’s critical values with all items identified as relevant, clear, and representative of the intended constructs. This stringent rating provides confidence that the scale captures the multi-faceted nature of insect-related fear for the young population, an application which adheres with the MEASURE framework for the development of psychometrics [31]. A CVI of more than 0.79 denotes expert consensus that exceeded the standard of Polit and Beck (2006) [33]. Thus, the results of the present study indicate that the instrument correctly reflects the intended constructs and adheres to best practices for content validity for new questionnaire development [27].

Both the exploratory factor analysis (EFA) and the confirmatory factor analysis (CFA) were used to test construct validity. EFA extracted a clear four-factor solution for the conceptual factors: knowledge, entomophobia, pesticide behavior, and personal anxiety. All of the factor loadings were above the minimum recommended level of 0.40 [34]. CFA also validated this structure with fit indicators CFI and TLI above 0.90 and RMSEA and SRMR below 0.08. These are contained within generally accepted cut-off values suggested by [35], determining that the hypothesized four-factor structure provides an adequate fit with the data. The results thus determine that the scale has strong construct validity and factorial stability according to accepted guidelines for the test evaluation of psychological tests [27]. This contrasts with previous validation of related phobia instruments such as the Specific Phobia Questionnaire [36] and the Spider Phobia Questionnaire [9], which have similarly determined strong factorial validity.

Reliability analyses produced strong results. Cronbach’s alpha coefficients of the subscales and of the overall scale all surpassed 0.70, with most of them greater than 0.80. The Cronbach’s alpha of 0.70 is satisfactory for first-stage investigations, while values greater than 0.80 reflect good internal consistency [37]. The findings thus confirm that the scale exhibits strong internal consistency. These results are compatible with other validated scales of phobia and fear [38]. Moreover, test–retest reliability, tested for 103 children at an interval of two weeks, yielded intraclass correlation coefficients (ICC) greater than 0.80, which according to Koo and Li (2016) reflect outstanding temporal stability [39]. This means that the tool produces consistent scores on many occasions and can safely be utilized for cross-sectional and longitudinal investigations. These values are compatible with prior evidence of stable scores of well-constructed questionnaires of fear across time intervals [40]. Absence of appreciable floor and ceiling effects is another strength of this instrument. As per COSMIN guidelines, floor or ceiling effects are problematic if more than 15% of respondents affirm the lowest or highest possible score [41]. In the present study, no such concentration has been observed, which means that the scale possesses sufficient sensitivity to detect differences at divergent knowledge, insect-related fear, and children’s behaviors.

In comparison with existing instruments, the current questionnaire represents an advancement due to its capability not merely of assessing insect-related fear responses emotionally and physically but also of including cognitive and behavioral domains, namely knowledge and practices of handling pesticides. This broader conceptualization has relevance for those situations for which children’s coping styles and health practices are potentially confounded with their insect-related fear responses and for which existing instruments such as the Specific Phobia Questionnaire [36] and the Spider Phobia Questionnaire [9], have tended to omit and concentrate on the purely affective and physiological responses. In addition, the assessment of knowledge and behavioral domains simultaneously results in an assessment that has higher ecological validity and higher cross-cultural appropriateness, particularly for those societies for which interaction with insects and the use of pesticides belong to their experience [42].

This study has several strengths. Utilizing a large and representable sample of 1,370 children, which has been selected from all education levels and counties by means of multi-stage stratified random sampling, enhances the generalizability of results. Utilizing an intensive psychometric system with content validity indices, EFA, CFA, test–retest analyses, and reliability further enhances methodological rigor. The use of cognitive, behavioral, and emotional characteristics of entomophobia also provides a holistic evaluation compared with previous instruments.

Nonetheless, there are also some recognized limitations. First, the sample included a wide age range of children. Although supplementary analyses indicated that age had only negligible associations with the construct scores, developmental differences may still exist and could be explored more comprehensively in future research using age-stratified analyses or measurement invariance testing. Second, the gender distribution of the sample was unbalanced, with a larger proportion of female participants. Despite the minimal gender differences observed in construct scores, future studies are encouraged to recruit more balanced samples or apply multigroup analytical approaches to examine potential gender-based variations in measurement properties. There are possibilities of response bias with use of self-report questionnaires since respondents may end up exaggerating or underestimating their experience. The sample consisted of only one of the provinces of Iran, hence limiting generalizability to other ecological or cultural contexts; replication with other populations is essential. Although no validated Persian instrument exists for measuring insect-related fear or related behaviors in children, future studies may consider evaluating convergent validity by comparing the questionnaire with broader psychological constructs, such as general anxiety or environmental fear scales. This study did not also test criterion validity through correlation with clinical diagnosis, observed behavior, or fear physiological markers and this would further establish the usefulness of the measure. COSMIN guidelines recommend for future validation studies that these concerns are resolved as steps towards augmenting the evidence base for this measure [27].

In general, findings endorse the value of the measure as a valid and reliable measure of insect-related fear and associated behaviors for participants of school age. They imply broad implications for public health as well as education interventions. Teachers, health workers, and counselors can utilize the measure as an assessment of participants with heightened insect-related fear, for developing coping responses, and for building education initiatives on appropriate and effective pest control. Future studies should maintain validation efforts with diverse populations, explore predictive validity with scores and clinical/behavioral outcomes, and explore test-sensitivity of the measure with intervention studies.

## Conclusion

Overall, this study developed and validated a novel questionnaire to measure knowledge, insect-related fear, pesticide-related behavior, and anxiety among school-aged children. The measure had high content validity, construct validity, internal consistency, and test–retest stability without floor or ceiling effects. Using cognitive, behavioral, and affective domains it fulfills an essential gap in children’s entomophobia assessment. It is a valuable tool for researchers as it is for practitioners with potential use in psychological measurement, programing in schools, and health educational intervention. With further validation in a broad range of populations in an effort based on demography and culture, the questionnaire has a potential for becoming a final measure of fear towards insects for use in- and out-of-laboratory applications.

## Supporting information

S1 Table Questionnaire for assessing Insect phobia in Iranian children-aged group. (DOCX)

S1 Dataset The study dataset. (SAV)
