# Supplementary material for: Development and psychometric validation of the insect fear questionnaire for school-aged children in Iran
Source: PLoS One. 2026 Mar 6;21(3):e0344126. doi: 10.1371/journal.pone.0344126 (PMC12965557; doi:10.1371/journal.pone.0344126)
Supplement: S1 Table — (DOCX) [file pone.0344126.s001.docx]

**S1 Table.** Questionnaire for assessing Insect phobia in Iranian children-aged group

| **Item** | **Answer** | | | | |
| --- | --- | --- | --- | --- | --- |
| **Knowledge** | **Strongly disagree** | **Disagree** | **No Idea** | **Agree** | **Strongly agree** |
| Q1. I can easily identify insects and spiders. |  |  |  |  |  |
| Q2. I am familiar with how insects and spiders live. |  |  |  |  |  |
| Q3. Insects and spiders are useful creatures in nature. |  |  |  |  |  |
| Q4. Insects and spiders cause allergies in some people. |  |  |  |  |  |
| **Entomophobia** | **Strongly disagree** | **Disagree** | **No Idea** | **Agree** | **Strongly agree** |
| Q5. I get scared if I see an insect or a spider. |  |  |  |  |  |
| Q6. If I see an insect or a spider, I think it will jump on me. |  |  |  |  |  |
| Q7. I am afraid of insect and spider bites. |  |  |  |  |  |
| Q8. I hate insects and spiders. |  |  |  |  |  |
| Q9. Insects and spiders are one of my worst fears. |  |  |  |  |  |
| Q10. I feel uncomfortable if I see a picture of an insect or a spider. |  |  |  |  |  |
| Q11. If I see an insect or a spider, I can hardly stay calm. |  |  |  |  |  |
| Q12. I'm worried that there are a lot of insect and spider nests around me. |  |  |  |  |  |
| Q13. I get worried if someone says there are insects or spiders around me. |  |  |  |  |  |
| Q14. I am very worried that I may be allergic to insect and spider bites or stings. |  |  |  |  |  |
| Q15. If I hear the sound of an insect, I become extremely anxious. |  |  |  |  |  |
| Q16. If I see an insect or a spider, I sweat with fear. |  |  |  |  |  |
| Q17. If I see an insect or a spider, I run away. |  |  |  |  |  |
| Q18. Before I enter a room, I check to make sure there are no insects or spiders there. |  |  |  |  |  |
| Q19. If I see an insect or a spider, I can't get it out of my mind for a long time. |  |  |  |  |  |
| Q20. If I have seen insects or spiders in a room before, I am worried about entering that room. |  |  |  |  |  |
| Q21. If I see an insect or a spider, I ask someone to kill it. |  |  |  |  |  |
| Q22. If I see an insect or spider in a room, I quickly leave the room. |  |  |  |  |  |
| Q23. Even if there are insects and spiders in the neighbor's house, I will do my best to get rid of them. |  |  |  |  |  |
| **Behavior** | **Never** | **Rarely** | **Sometimes** | **Often** | **Always** |
| Q24. I use insect repellent spray or gel on my skin. |  |  |  |  |  |
| Q25. I use insecticides at home. |  |  |  |  |  |
| Q26. I use insecticides at school. |  |  |  |  |  |
| Q27. I have insecticide with me. |  |  |  |  |  |
| **Personal fear and Anxiety** | **Never** | **Rarely** | **Sometimes** | **Often** | **Always** |
| Q28. I am a worrier and feel anxious about everything. |  |  |  |  |  |
| Q29. When I have a problem, I get a strange feeling in my heart. |  |  |  |  |  |
| Q30. I am a fearful person. |  |  |  |  |  |
| Q31. Whenever I am worried or stressed, my heart beats fast. |  |  |  |  |  |
| Q32. I'm worried that something bad will happen to me. |  |  |  |  |  |
